# Living myocardial slices: walking the path towards standardization

**DOI:** 10.1093/cvr/cvaf079

**Published:** 2025-05-12

**Authors:** Jort S A van der Geest, Teun P de Boer, Cesare M Terracciano, Thomas Thum, Andreas Dendorfer, Pieter A Doevendans, Linda W van Laake, Joost P G Sluijter, Vasco Sampaio-Pinto

**Affiliations:** Experimental Cardiology, Department of Cardiology, University Medical Center Utrecht , Heidelberglaan 100, 3508 GA, Utrecht, the Netherlands; Regenerative Medicine Center Utrecht, Circulatory Health Research Center, University Utrecht, Uppsalalaan 8, 3584 CT, Utrecht, the Netherlands; Department of Medical Physiology, Division of Heart & Lungs, University Medical Center Utrecht, Yalelaan 50, 3584 CM, Utrecht, The Netherlands; National Heart & Lung Institute, Imperial College London, 72 Du Cane Road, W12 0NN London, UK; Institute of Molecular and Translational Therapeutic Strategies (IMTTS), Hannover Medical School, Carl-Neuberg-Straße 1, 30625 Hannover, Germany; Walter-Brendel-Centre of Experimental Medicine, University Hospital, German Center for Cardiovascular Research (DZHK), Munich Heart Alliance (MHA), LMU Munich, Marchioninistraße 27, 81377 Munich, Germany; Experimental Cardiology, Department of Cardiology, University Medical Center Utrecht , Heidelberglaan 100, 3508 GA, Utrecht, the Netherlands; Netherlands Heart Institute (NLHI), NT-TESTMoreelsepark 1, 3511 EP, Utrecht, the Netherlands; Central Military Hospital (CMH), Lundlaan 1, 3584 EZ, Utrecht, the Netherlands; Experimental Cardiology, Department of Cardiology, University Medical Center Utrecht , Heidelberglaan 100, 3508 GA, Utrecht, the Netherlands; Regenerative Medicine Center Utrecht, Circulatory Health Research Center, University Utrecht, Uppsalalaan 8, 3584 CT, Utrecht, the Netherlands; Experimental Cardiology, Department of Cardiology, University Medical Center Utrecht , Heidelberglaan 100, 3508 GA, Utrecht, the Netherlands; Regenerative Medicine Center Utrecht, Circulatory Health Research Center, University Utrecht, Uppsalalaan 8, 3584 CT, Utrecht, the Netherlands; Experimental Cardiology, Department of Cardiology, University Medical Center Utrecht , Heidelberglaan 100, 3508 GA, Utrecht, the Netherlands; Regenerative Medicine Center Utrecht, Circulatory Health Research Center, University Utrecht, Uppsalalaan 8, 3584 CT, Utrecht, the Netherlands

**Keywords:** Living myocardial slices, Disease modeling, Translational research, Mechanical and electrical stimulation, Metabolism

## Abstract

Cardiovascular disease remains a persistent global health burden, underscoring the necessity for effective therapeutic strategies. Despite significant advances, the ability to mechanistically study human disease and predict clinical outcomes remains limited, especially in complex diseases such as heart failure. This limitation is evident through the continuous high attrition rates in drug development pipelines. To address these challenges and contribute to improved preclinical studies, there is a need for platforms that more accurately recapitulate the human heart. This need increased the interest in living myocardial slices (LMS) — thin sections of the heart of approximately 100–400 μm. LMS retain the native multicellular architecture of the heart and enable extended *ex vivo* culture. However, as their utilization grows, so does variability in preparation methodologies and readouts. This review provides an overview of differences in sample selection, interspecies variations, intra-cardiac differences, and potential confounding factors. Additionally, we examine culture methods, addressing electrical and mechanical stimulation differences, and medium compositions. Our review concludes by highlighting the current limitations of LMS research and offers guidelines for standardization and future applications. The ultimate aim of this review is to serve as a resource for researchers working with LMS and for those entering this field. By presenting the landscape of methodological considerations, we aim to facilitate informed decision-making in study design and execution. We advocate for accurate reporting of methodologies to promote reproducibility and comparability across studies, advancing LMS research and strengthening its role as a valuable addition to the current drug development toolbox and basic cardiovascular research.

## Graphical Abstract

**Graphical Abstract:**
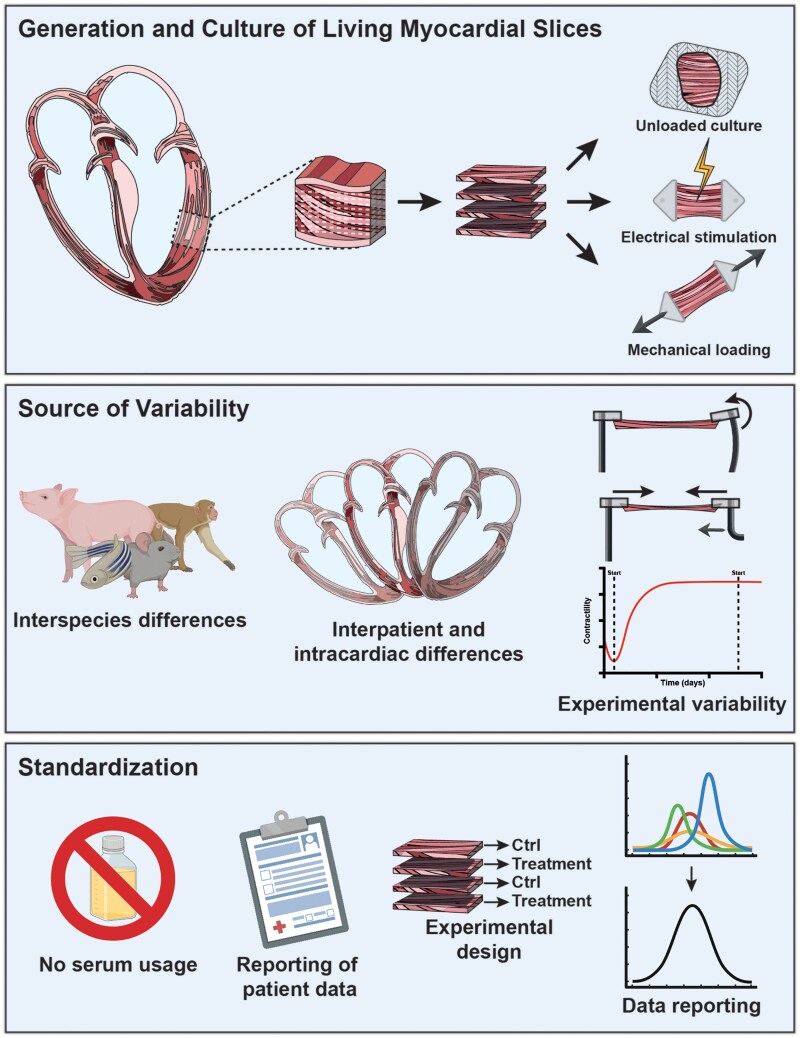
Living myocardial slices (LMS) have become a valuable preclinical model to study cardiac (patho)physiology and therapeutic interventions. However, the rise of studies employing LMS has come with an increased experimental and methodological variation. Unlocking the full potential of this model requires standardization.

## 1. Introduction

### 1.1 Challenges in cardiovascular drug development

Over the past decades, medical advancements have extended the life expectancy of individuals with cardiovascular diseases.^1^ However, the chronic and progressive nature of these conditions continues to pose a growing economic and societal burden,^2^ highlighting the need for novel cardiovascular drugs. Alarmingly, only 1 in 20 new cardiovascular medicines that enter clinical development progress to reach clinical application,^3^ primarily due to a lack of efficacy and unwanted drug-related toxicity.^4^ Cardiotoxicity is one of the three main adverse drug reactions,^5^ hampering the development of both cardiovascular and non-cardiovascular medicines. Notably, the presence of non-cardiomyocytes in the heart is thought to play an important role in drug-induced cardiac toxicity.^5^ The complexity of the cardiovascular system and the multifactorial nature of many cardiovascular diseases hinder the development of predictive preclinical cardiac models. Current preclinical models, including animal studies and isolated cardiomyocytes, often fail to accurately predict human responses due to species differences and lack of physiological complexity, respectively. While advancements like human-induced pluripotent stem cell-derived cardiomyocytes (hiPSC-CMs) and generation of engineered heart tissues (EHTs) have allowed for patient-specific studies and high-throughput testing, it remains difficult to mimic the architecture and multicellularity seen in the native myocardium. Historically, papillary muscle and trabeculae preparations helped build our understanding of cardiac physiology. However, while they preserve cardiac tissue architecture and multicellularity, their endocardial origin precludes a good representation of the myocardium. In addition, these preparations are often above 400 μm thick, resulting in poor oxygenation, formation of a necrotic core, and limited viability and longevity.^6^ Moreover, the chronic nature of cardiovascular diseases, characterized by years of pathological remodeling and high heterogeneity between patients, is hard to mimic in a laboratory setting. Consequently, these factors make the current preclinical pipeline unsatisfactory in accurately replicating the human condition, highlighting the need for improved human-based models. Living myocardial slices (LMS) can overcome these limitations by retaining the native multicellular architecture of the heart, capturing the accumulation of pathological remodeling and the high patient-to-patient variability seen in the human population.

### 1.2 A brief history of LMS

LMS have emerged as a promising approach to bridge the gap between preclinical testing and clinical application, often referred to as the ‘valley of death’ of drug development. The first report of LMS dates back to 1946,^7^ but since then, the field has experienced rapid growth with diverse technical approaches and applications. The first studies employing LMS relied on hand-held blades to generate the slices.^7,8^ These early studies required a high level of skill and were limited by the difficulty in consistently generating slices without significant tissue damage. Technical advancement was obtained by the introduction of high-precision vibratomes, thereby minimizing myofiber transection during slicing, enhancing slice viability, and increasing reproducibility.^9^ Early applications of LMS were diverse but focused on acute studies due to difficulties in maintaining tissue functionality over time. Recognizing the necessity to preserve LMS functionality for extended periods, researchers developed initial culturing methods such as fully submerging the slices in media,^9–12^ using a liquid-air interface, such as a transwell system,^13,14^ or in-house designed pillar array.^15^ However, these primordial culturing methods were limited in their ability to preserve the function and structure of the tissue beyond hours to several days, with the myocardial phenotype being lost due to irreversible dedifferentiation.^16^

**Figure 1:**
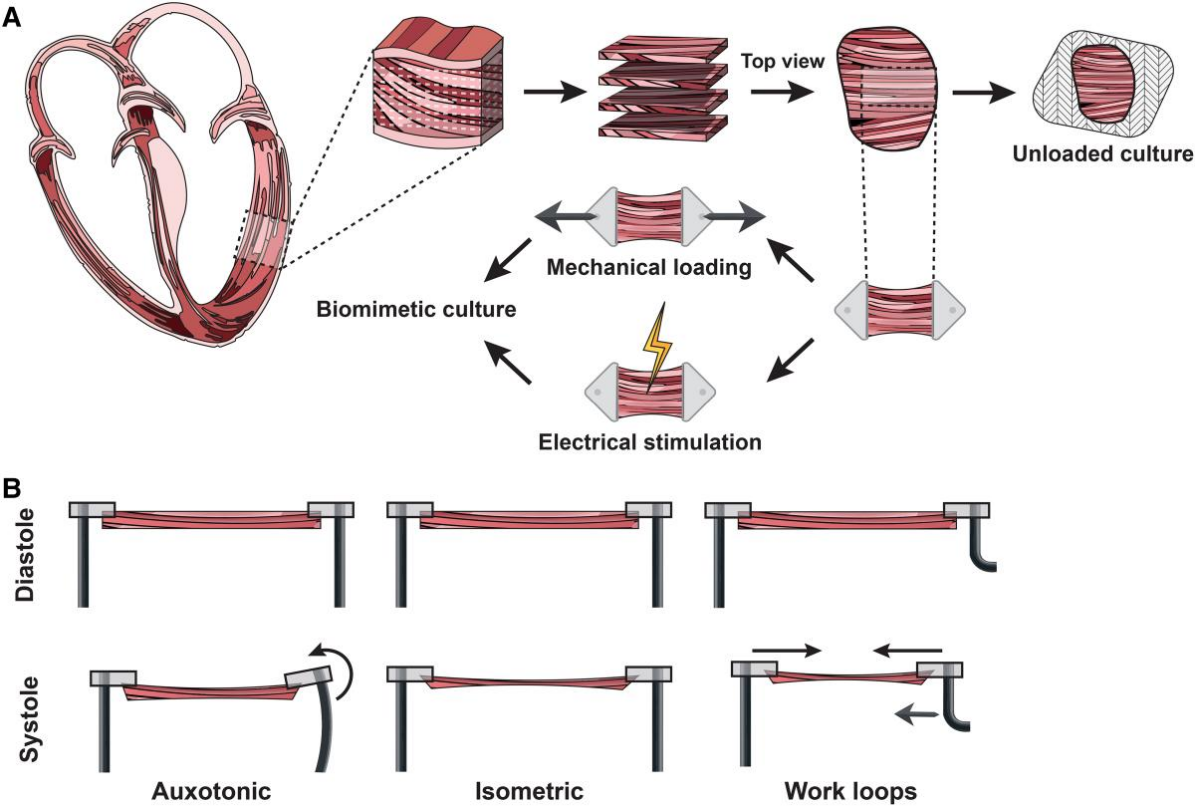
Overview of LMS preparation and culture conditions: *A*) Schematic representation of LMS generation from a ventricular biopsy, highlighting the subsequent culture setup with options for mechanical loading and electrical stimulation. *B*) Illustration of three distinct mechanical loading methods applied to LMS: Auxotonic, utilizing flexible poles to create a variable afterload; Isometric, with fixed poles resulting in a maximal, continuous afterload; and work loops, where an actuator adjusts length, offering the most accurate representation of natural cardiac mechanics.

To overcome these limitations and thus extend the myocardial phenotype of LMS in time, efforts were made to provide culture conditions that more closely resemble the environment *in vivo*. The addition of electrical pacing to LMS culture extended the culture period up to six days while preserving function and transcriptomic status.^17,18^ Similarly, applying mechanical load during culture allowed sustained contractions for up to seven days post-slice preparation.^19^ Combining mechanical loading and electrical pacing set the advent of biomimetic culture systems, establishing a platform for chronic studies of up to 4 months in culture while maintaining (patho)physiological properties of LMS.^20^ An overview of the workflow of generating LMS and the various culture conditions is shown in *Figure 1*. These advancements have significantly enhanced the utility of LMS, allowing for more accurate modeling of cardiac function and disease over extended periods. Recent improvements in media composition, including the addition of specific hormones and growth factors, have further enhanced the ability to maintain LMS viability and function.

In light of the increasing focus on animal-free experimentation and the goal of achieving clinical approval through completely animal-free preclinical trajectories, there is growing interest in generating LMS from human myocardial samples. This approach enhances the translational potential of preclinical research and is likely to improve predictions of drug toxicity. This review aims to provide a guideline for informed decision-making when utilizing LMS, explaining the different systems used, the improvements achieved thus far, and their implications. By emphasizing considerations on sample selection, culturing methods, reporting, and analysis, we aim to reduce variability, increase reproducibility, and foster coherence, and standardization in the field.

## 2. Sample selection

### 2.1 Interspecies differences

**Figure 2:**
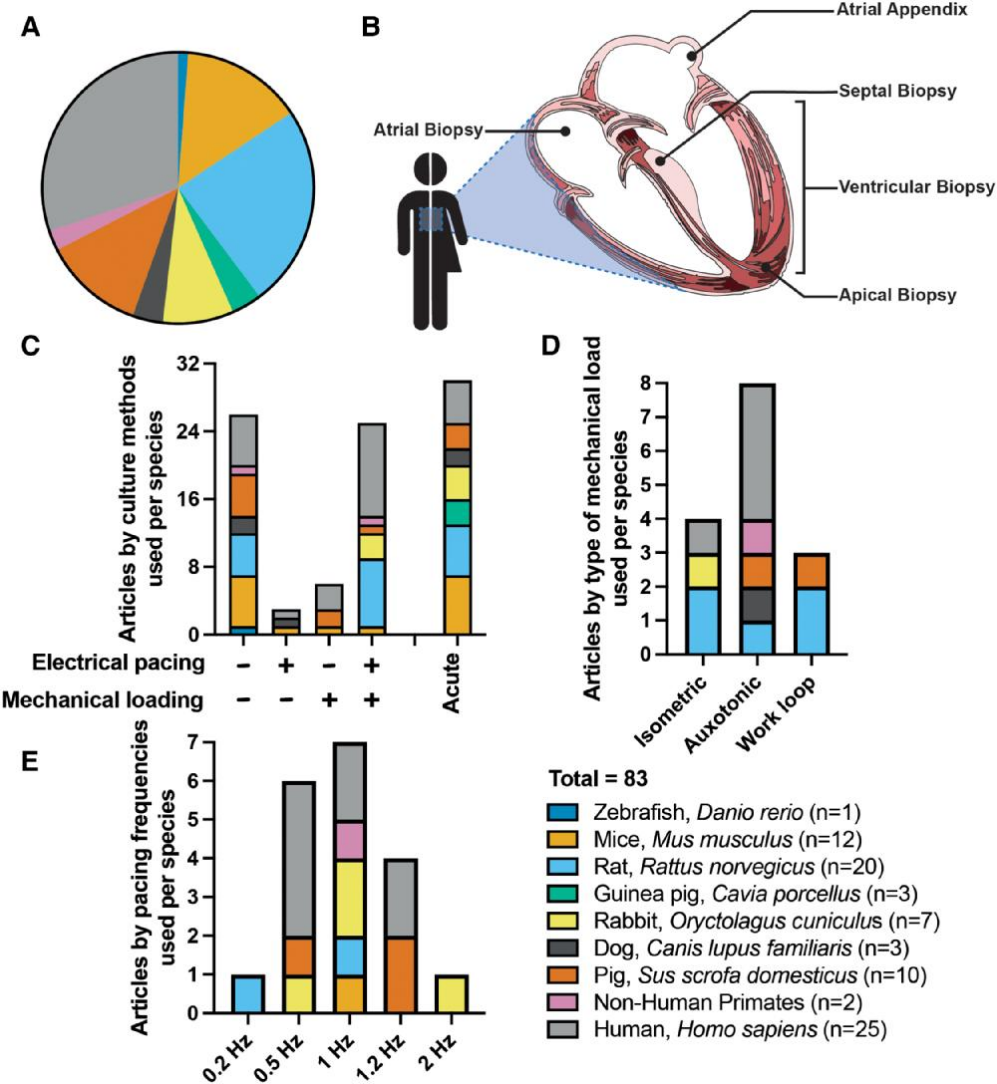
Overview of LMS studies: species sources, intra-cardiac sample selection, and culture conditions. A PubMed search using the term “myocardial slice” was conducted. A total of 83 articles were included in this analysis, corresponding to studies reporting the preparation of LMS from different animal models and, when available, the employed culture conditions. *A*) Among these publications, nine different species were used to produce LMS. *B*) For human-based studies, there are five different intra-cardiac locations used to produce LMS: ventricles, atria, atrial appendices, LV apex, and septal myectomies. The figure illustrates the wide variability in culturing methodologies across these species, encompassing differences in mechanical loading (*C* & *D*) and electrical stimulation (*C* & *E*). This heterogeneity highlights the lack of standardized protocols in the field and underscores the necessity for standardization in myocardial slice research.

Preserving optimal viability and cardiac tissue functionality is indisputably pivotal for any LMS-based study. Yet, it is important to consider that interspecies differences may influence study designs, experimental outcomes, and translational potential. Given the wide array of small and large model organisms, shown in *Figure 2*, from which LMS have been derived, important factors such as differences in electrophysiological, physiological, metabolic, and proliferative capacity properties require careful consideration.

Interspecies differences manifest in electrophysiological properties, such as variations in action potential durations and propagation speeds, primarily due to alterations in ion channel expression and cell-to-cell connections.^21,22^ These electrophysiological differences lead to diverse baseline sinus rates across species; while humans exhibit a resting heart rate of 60–100 beats per minute (bpm), mice demonstrate a resting heart rate of approximately 500 bpm. These mechanical and electrical differences have been considered for LMS culture, human LMS are typically paced between 0.2 and 1.2 Hz,^14,16,18,20,23^ while rodents at 1 up to 2 Hz,^16,24,25^ as displayed in *Figure 2C* and *2E*. However, recent literature indicates that culturing rabbit LMS above 1 Hz results in a progressive decline in contractile force.^24^ While this decline could be attributed to the depletion of the media or a redox shift due to pacing, this has not been empirically demonstrated.

While substrate preferences among species are generally similar, ionic balance might be achieved differently between organisms. Specifically, rodents differ in ionic balance and electrophysiological response from humans, whereas dogs and rabbits show more similarity,^26^ for example, the sarcoplasmic/endoplasmic reticulum Ca^2+^-ATPase (SERCA) pump removes 90% of cytosolic calcium in rodents, while for rabbits and humans, it is around 70%.^27^

Beyond differences in cardiomyocytes, interspecies variations extend to the differences in cellular composition, where humans tend to have a higher fibroblast to cardiomyocyte ratio, and mice seem to have a denser vascularization and more resident immune cells.^28–30^ Additionally, extracellular matrix (ECM) composition and structure contribute to distinct mechanical properties, leading to variations in contraction dynamics and overall tissue behavior between species. While LMS retain the native tissue architecture and ECM compared to single cells, dedifferentiation remains a problem. Dedifferentiation is especially evident in smaller rodents due to the higher metabolic needs.^16^ Even though interspecies differences create additional challenges to LMS research and limit translation to humans, they also offer unique opportunities. LMS derived from zebrafish retain regenerative capacity and, therefore, offer a platform for studying and modulating cardiac regeneration.^10^ Similarly, embryonic human ventricles were used to investigate the regeneration of the human heart.^31^

It is important to note that interspecies differences not only have their obvious effects on the outcome, and the translatability of the results, but also determine the culture conditions. Empirical studies are required to determine the most adequate conditions for LMS culture of different species, which will eventually enhance the translatability of findings from animal models to human clinical applications.

### 2.2 Inter- and intra-cardiac differences

In this section we focus on human tissue but it is worth mentioning that the same considerations are valid for other species. Samples obtained from human sources include deceased donor cardiac tissue,^14,23^ ventricles^16,19,23,32,33^ and atria^34^ of explanted hearts, small ventricular biopsies,^35,36^ septal wall obtained during a myectomy^32,37–40^ atrial appendices^34^  ^,41^ and left ventricular apex tissue obtained after LVAD implantation.^40,42–44^ The location of the tissue within the heart significantly impacts the characteristics of LMS. For example, ventricular tissue typically has a higher density of cardiomyocytes and generates greater contractile force compared to atrial tissue due to differences in workload and function.^45^ Atrial tissue also exhibits distinct electrophysiological properties, such as shorter action potential duration and different ion channel expression, compared to ventricular tissue.^46^ Additionally, the alignment of myofibers varies throughout the heart; in the ventricles, myofiber orientation changes gradually from the endocardium to the epicardium, forming a complex helical structure. This structure poses challenges in preparing LMS with consistent fiber alignment, which is crucial for their mechanical properties, longevity, and force generation, more evident in atrial tissue.^20,33^ In the disease state, procurement of myocardial tissue from certain cardiac regions may also result in technical differences. The increased presence of fibrosis, more likely to occur in a diseased state and towards the epicardium, can lead to challenges in consistently producing LMS. These intricate differences extend to transmural variations, where LMS of rats showed that a greater surface area is covered by cardiomyocytes towards the epicardium, resulting in a stronger passive and active tension.^47^ This transmural gradient is exacerbated in a diseased state. To date, the majority of studies involving human LMS have originated from diseased specimens; however, critical factors like etiology, genetic background, co-morbidities, and drug usage often remain under-reported. In Section 4, we discuss current gaps in LMS culture, delve into the possible implications of these factors and highlight current knowledge gaps in LMS research.

## 3. Culturing LMS

The heart, the first terminally differentiated and functional organ in mammals, orchestrates its complex functions through an interplay of metabolic, hormonal, mechanical, and electrical stimulation. While these processes are inextricably linked, we discuss the importance of these factors during the culturing of LMS. Given the heart's remarkable adaptive capabilities, LMS swiftly respond to environmental changes. It is therefore imperative that the native cardiac conditions are mimicked upon culture to maintain LMS viability and function and prevent unwanted alterations.

Initial attempts to culture LMS employed methods such as a liquid-air interface on a transwell system or submersion in media in an orbital shaker.^14,33,48^ However, the absence of mechanical loading and electrical stimulation in these initial studies resulted in the dedifferentiation of cardiomyocytes, as evidenced by a rapid loss of sarcomere and t-tubule structures, loss of cell-cell connections seen by connexin density^16,18^ and increased proliferation of fibroblasts.^19^ The advent of biomimetic culture systems, that more closely resemble the state of the native heart, prolonged LMS usage by preserving calcium handling and contractile function upon long-term culture.^16,20^

### 3.1 Mechanical loading in culturing LMS

As evidenced during the unloaded culture of LMS, mechanical loading is an important regulator of cardiac structure and function. In a normal range of mechanical load, structural changes are prevented and LMS display optimal cardiac function. Instead, chronic mechanical unloading has been shown to result in atrophy,^16,49^ whereas chronic overloading leads to hypertrophic cardiomyocyte dysfunction and the development of heart failure.^16^ Interestingly, molecular signatures of overloading and unloading seem to reactivate part of fetal gene programs as a survival program, including metabolic and functional rewiring.^50,51^ This suggests that the range of physiological loading may be dynamic and changes throughout life. In the fetal development of the heart, increased loading is a driving force for the development of the adult heart as we know it^52,53^ while in a failing heart, mechanical unloading of an overloaded heart by a left ventricular-assisted device (LVAD) may restore the heart structurally and, in limited cases, even functionally.^54^

Considering the crucial role of mechanical load for culturing LMS, culturing end-stage heart failure tissue in a state of physiological loading may prompt reverse remodeling towards a healthier state. Conversely, overloading healthy LMS has been shown to promote adverse remodeling, in line with the development of a heart failure phenotype.^16^ In the 3D heart, mechanical forces are complex. Preload corresponds to the mechanical force that the heart is exposed to in the relaxed diastolic phase, while the afterload relates to the force required to pump blood into the circulation. In LMS culturing, these concepts were also considered and rather simplified, resulting in uniaxial isometric and auxotonic loading strategies, both applying a desired preload by stretching the tissue on a fixed^16,48^ and flexible pole,^20^ respectively (*Figure 1*). While isometric loading results in a maximal, continuous afterload, auxotonic loading results in a variable afterload. For the latter, the resistance of the flexible pole should be adjusted so that the tissue is allowed to shorten within physiological ranges. Importantly, the preload of LMS can be measured based on sarcomere length, (*i.e.* distance between two adjacent z-disks) and can be set to a physiologic (1.8–2.2 μm) or a pathophysiologic range (>2.2 μm). However, measuring sarcomere length in slices is not trivial, therefore to expedite preload establishment, two strategies have been proposed and validated: (i) geometrically setting the preload with the assistance of a caliper by measuring the length of the unloaded LMS and stretching the slice lengthwise to a fixed percentage^16,19,55^ or (ii) by promoting a stretch of the LMS lengthwise until a diastolic tension between 0.3 and 1.5 mN is achieved.^20,24,25,56,57^ For the latter, tension re-adjustment is advised 24 h after the start of culture and at each media exchange (48–72 h intervals) during the first week of culture. While these two strategies and their implications have not been systematically compared, both have shown to successfully stretch slices to a physiologic preload (*i.e.* sarcomeric length between 1.8 and 2.2 μm) and allow culture for multiple days.^20,55^ The first strategy relies on the use of a caliper, which may inadvertently lead to inaccuracies while the second approach relies on the use of proprietary hardware and software allowing real-time assessment of tension. Hence, while the second approach can be deemed more accurate and user-friendly, the first may still be used in the absence of such culture systems. Modeling dynamic conformational changes for a more accurate representation of the cardiac cycle requires a work loop in which an actuator allows for changing the length of the slice.^58–60^ In this elegant model, pre- and afterload can be manipulated separately to mimic more accurately advanced diseases like pressure- and volume-overload. Work loops have both been simulated by a fixed stretch protocol and by dynamically adjusting the length based on real-time contractile force, known as a feed-forward^60^ and feedback systems,^58^ respectively. Though dynamically adjusting the length allows for a better representation of the pre- and after-load of the heart, these approaches still oversimplify the heart's complex mechanics by imposing uniaxial loading. Alternatively, employing 3D electromechanical stimulation using pressurized silicon membranes aims to more accurately replicate the 3D physiological stretching in LMS culturing.^61^ However, while the heart's stretch is not uniaxial, it is also not equal in all directions due to the cardiac anisotropic fiber architecture, as illustrated in *Figure 1A*. The method described provides uniform stretching in all directions, which does not fully capture the varying degrees of stretch experienced *in vivo*. Additionally, this method^61^ is limited by deriving contractile force using video-assisted strain analysis, which indirectly converts the observed displacement into force, assuming that stiffness is homogeneous within the slice and between different slices, thus being less accurate. Overall, substantial efforts have been made to mimic the mechanical conditions of the heart, but comparative studies evaluating LMS cultured in different loading strategies against fresh uncultured tissue are generally lacking. Additionally, the potential benefit of the increased complexity of the dynamic stretching approach compared to simpler uniaxial loading strategies still needs to be demonstrated through comparative studies. While dynamic stretching may provide a more accurate representation of the heart's 3D mechanical environment, the added complexity, potential lower throughput, and challenges in implementation could limit its practical application. Further research is required to evaluate whether the improved physiological relevance of dynamic stretching translates into meaningful advantages for understanding cardiac function and therapeutic evaluations.

### 3.2 Electrical stimulation in culturing LMS

Contraction is a highly coordinated process that is controlled by a complex network of ion currents within and between cells. In contrast to direct cell-to-cell propagation observed in the heart, cultured LMS are stimulated using field stimulation, where the electrical stimuli are released from an electrode to the media and reach all the cells within the slice simultaneously, subsequently inducing the contraction. This is due to practical limitations in establishing actual point stimulation using electrode stimulation on a beating slice. Moreover, electrical stimulation in LMS culture ranges from a subphysiological to resting rate, typically ranging from 0.2 Hz^20^ to 1.2 Hz^61^ for human tissue (*Figure 2E*). This relatively slow pacing aims to preserve tissue integrity and limit media composition alterations caused by metabolic depletion and continuous pacing-induced redox shifts. The long-term effects of varying beating rates in LMS cultures remain largely unexplored, and comparisons across methodologies are challenging due to considerable variations in approaches. Notably, the artificial pacing strategies lack heart rate variability, a crucial marker inversely correlated with cardiovascular age and health.^62^

Exploring alternative pacing protocols could offer valuable insights. For instance, intricate processes like tachycardia can be mimicked by generating a train of fast pulses, which can also inform about the slices' susceptibility to arrhythmogenic pacing.^63,64^ Similarly, simulating exercise might present an intriguing avenue, in which the effect of chronic exercise can finally be researched in a controlled environment. This could be achieved utilizing either a patterned stimulation protocol between fast and slow pacing, or an adaptive stimulation protocol wherein contractile force drives the pacing to closely mimic the dynamic process of exercise.

### 3.3 Metabolic and hormonal considerations in culturing LMS

Contraction and ion homeostasis demands high metabolic activity. A healthy adult human heart typically has a turnover of approximately 6 kg of ATP per day,^65^ primarily produced through oxidative phosphorylation using lipid substrates.^66^ In a prenatal state, however, glycolysis is the main source of ATP production.^67^ The main metabolic substrate used is driven by hormones, where thyroid hormones increase oxidative phosphorylation and insulin, glucocorticoids, and catecholamines increase glycolysis.^68^ In hiPSC-CMs, it has been shown that a switch from a glucose-rich medium to a fatty-acid-rich medium enhanced maturation.^69,70^ While the healthy heart exhibits great metabolic flexibility, this adaptability is lost under pathological conditions, leading to a reversion to glycolysis.^71^

The specific metabolic requirements for LMS cultures remain relatively unknown and are dependent on the tissue's state and species. The variety of basal media, antibiotics, and supplements reported thus far for LMS culture are given in *Table 1*. The most commonly used culture medium for LMS is Medium-199, a glucose-rich media, supplemented with insulin, transferrin, and selenite (ITS). While this basic medium sustains basal cardiac viability, findings regarding its adequacy after electrical stimulation vary. Reports show a rapid decline in viability and contractility at pacing rates of 1.2 to 2 Hz,^18,24^ whereas studies with lower pacing rates indicate sufficient support for viability following stimulation.^16^

**Table 1:** Media compositions used for LMS culture

Medium base	Supplements	Concentration range	Culture Conditions (Electrical pacing/Mechanical loading)	Species	Ref
Claycomb medium	+ noradrenaline	100 mM	−/–	Mice, *Mus musculus*	^13^
	+ FBS	10%			
	+ L-glutamine	4 mM			
DMEM	−	−	−/–	Mice, *Mus musculus*	^72^
DMEM 1:1 F12	−	−	+/+	Rat, *Rattus norvegicus*	^58^
	+ knockout serum replacement	20%	−/–	Rat, *Rattus norvegicus* Human, *Homo sapiens*	^31^
	+ non-essential amino acids	1%			
	+ L-glutamine	2 mM			
	+ β-mercaptoethanol	0.1%			
	+ Pen/Strep	0.1%			
	+ FBS	10%	−/–	Mice, *Mus musculus*	^12^
	+ minimum essential medium non-essential amino acids	1%			
	+ L-glutamine	2 mM			
	+ Pen/Strep	1%			
DMEM + 10% Medium-199	+ horse serum	10%	−/–	Rat, *Rattus norvegicus*	^73^
	+ FBS	5%			
	+ Pen/Strep	1%			
IMDM	+ FCS	20%	−/–	Mice, *Mus musculus*	^74,75^
Krebs-Henseleit buffer	−	−	+/+	Rat, *Rattus norvegicus*	^58^
L15	+ Glutamax	1 ×	−/–	Zebrafish, *Danio rerio*	^10^
	+ BDM	20 mM			
	+ FBS	10%			
	+ Primocin	100 µg/mL			
	+ Pen/Strep	1%			
Medium-199 (Earle's salt)	−	−	+/+	Human, *Homo sapiens*	^76^
	+ ITS + Pen/Strep	1 × 1–3%	−/− +/− −/+ +/+	Mice, *Mus musculus* Rat, *Rattus norvegicus* Rabbit, *Oryctolagus cuniculus* Dog, *Canis lupus familiaris* Pig, *Sus scrofa domesticus* Human, *Homo sapiens*	^16,18,19,25,33,38,40,55,76–78^
	+ ITS	1 ×	−/–	Pig, *Sus scrofa domesticus* Human, *Homo sapiens*	^18,79^
	+ Pen/Strep	1–2%			
	+ BDM	10 mM			
	+ ITS + Pen/Strep + β-mercaptoethanol	1–5 × 1–5% 50 µM	−/− +/− −/+ +/+	Mice, *Mus musculus* Rabbit, *Oryctolagus cuniculus* Non-human Primate Human, *Homo sapiens*	^20,25,32,34,37,41,42,56,57,77^
	+ ITS + Pen/Strep + β-mercaptoethanol + cortisol	1 × 1–3% 50 µM 20–50 nM	+/+	Pig, *Sus scrofa domesticus* Human, *Homo sapiens*	^80,81^
	+ ITS + Pen/Strep + β-mercaptoethanol or cortisol or T3 or BDM or CytoD or blebbistatin or BTS or denopamine + salbutamol + phenylephrine	1 × 1% 50 µM 20 nM 10 pM 30 mM 5 µM 50 µM 50 µM 50 nM 50 nM 50 nM	+/+	Rabbit, *Oryctolagus cuniculus*	^24^
	+ ITS	1 ×	−/–	Dog, *Canis lupus familiaris*	^13^
	+ Pen/Strep	1%			
	+ BSA	0.1%			
	+ BDM	10 mM			
	+ chemically defined lipid	1×			
	+ HEPES	1×			
	+ ITS	1×	+/+	Rat, *Rattus norvegicus* Human, *Homo sapiens*	^23,82^
	+ Pen/Strep	2%			
	+ adrenaline	4 nM			
	+ noradrenaline	4 nM			
	+ dexamethasone	100 nM			
	+ T3	2.15 nM			
	+ ascorbic acid	20 µg/mL			
	+ ITS	1 ×	+/+	Rat, *Rattus norvegicus* Human, *Homo sapiens*	^83^
	+ Pen/Strep	2%			
	+ FBS	10%			
	+ adrenaline	4 nM			
	+ noradrenaline	4 nM			
	+ dexamethasone	100 nM			
	+ T3	2.15 nM			
	+ endothelial cell growth supplement	7.5 µg/mL			
	+ ascorbic acid	20 µg/mL			
	+ ITS + FBS + VEGF + FGF + antibiotic-antimycotic	1x 10% 5 ng/mL 10 ng/mL 2x	−/− +/− +/+	Pig, *Sus scrofa domesticus* Non-Human Primate Human, *Homo sapiens*	^14,17,18,61,84^
	+ adrenaline	4 nM	+/+	Rat, *Rattus norvegicus*	^59^
	+ noradrenaline	4 nM			
	+ dexamethasone	100 nM			
	+ T3	2.15 nM			
	+ ascorbic acid	20 µg/mL			
Medium-199 (Hank's salt)	+ ITS	1×	+/+	Rat, *Rattus norvegicus*	^85^
	+ adrenaline	4 nM			
	+ noradrenaline	4 nM			
	+ dexamethasone	100 nM			
	+ T3	2.15 nM			
	+ ascorbic acid	20 µg/mL			
	+ Pen/Strep	2%			
Waymouth's media	+ FBS	7.5%	−/–	Rat, *Rattus norvegicus*	^9^
	+ gentamicin	84 pg/mL			
	+ FBS	5.0%	−/–	Rat, *Rattus norvegicus*	^11^
	+ HEPES	25 mM			
	+ L-glutamine	2.4 mM			
	+ gentamicin	84 pg/mL			

BDM, 2,3-Butanedione monoxime; BSA, Bovine Serum Albumin; BTS, N-benzyl-p-toluene sulphonamide; CytoD, Cytochalasin D; DMEM, Dulbecco's Modified Eagle Medium; FBS, Fetal Bovine Serum; FCS, Fetal Calf Serum; FGF, Fibroblast Growth Factor; HEPES, 4-(2-hydroxyethyl)-1-piperazineethanesulfonic acid; IMDM, Iscove's Modified Dulbecco's Medium; ITS, Insulin-Transferrin-Selenium; Pen/Strep, Penicillin/Streptomycin; T3, Triiodothyronine; VEGF, Vascular Endothelial Growth Factor.

Medium-199 with Earle's salts is used to culture LMS under normoxic conditions, but to mimic a hypoxic condition similar to an acute ischemic response, Medium-199 with Hank's salts and HEPES has been used, with nitric oxide superfusion.^85^ This approach mimics the hypoxic environment observed during an acute ischemic event. Additionally, to model the infarct region, cryoinjuries can be conducted in the LMS.^10,86^ To further stimulate the fibrotic response, TGF-β can be supplemented to the culture medium.^86^

Preserving the heart's native state requires averting metabolic remodeling, particularly the decline in oxidative phosphorylation. It's worth noting that the failing heart adopts fetal-like metabolism as a protective measure^51^ and therefore the glucose-rich media composition might be beneficial for LMS of failing hearts. Conversely, LMS from healthy hearts acquired from animals or donors may require a lipid-rich media composition. Interestingly, the addition of lipids in culture media is limited^13^ and the supplementation with ketones, another common substrate in plasma, has to this day not been reported.

Apart from the nutritional content, another important change from the native heart is the absence of hormones and growth factors. A list of supplements added to LMS culture media is given in *Table 1*. This list consists of serum replacements, glucocorticoids, adrenergic agents, thyroid hormones, cytoskeletal inhibitors, and growth factors. Hormones play a crucial role in maintaining the native metabolic state of the heart and its integrity. Yet, caution needs to be paid to dosing, given that LMS lack the body's inherent negative feedback loop for hormone regulation. In more sophisticated systems, an ideal approach would involve implementing a feedback mechanism, *e.g.* triggering the release of noradrenaline or adrenaline into the medium in response to a decline in contractile force. This mechanism would better replicate physiological responses like exercise. While serum replacements such as fetal bovine serum (FBS) and horse serum provide essential nutrients, their variability, and undefined nature pose challenges to reproducibility and raise ethical concerns. The inconsistency in serum replacements can lead to unpredictable outcomes due to the nonspecific binding of compounds to serum proteins.

Another key change from *in vivo* to *ex vivo* is the absence of perfusion, the loss of shear stress, and hemodynamics, likely resulting in endothelial remodeling. The native heart's vasculature is constantly subjected to mechanical forces that shape endothelial cell function and vascular tone.^87,88^ In culture systems, adequate distribution of nutrients and oxygen can typically be achieved through either rocking or perfusion. It can be speculated that the pulsatile flow associated with perfusion more closely resembles *in vivo* conditions. However, the effects of culture methods on the vasculature have not been extensively studied. In addition, *in vivo,* endothelial cells are exposed to circulating immune cells, the coagulation system, and paracrine factors such as cytokines, growth factors, and metabolic signals. Instead, the contact of LMS with the immune compartment is restricted to tissue-resident leucocytes.^55^ This ‘clean’ LMS environment lacks paracrine factors and cellular interactions typically seen *in vivo*. Finally, when subjecting the medium to continuous electrical stimulation, the inclusion of a reducing agent, *i.e.* beta-mercaptoethanol or ascorbic acid to prevent a redox shift-induced oxidative stress is recommended.

In summary, culture media compositions vary substantially across studies, with a lack of systematic investigation into the acute and chronic effects of these formulations. Evaluating media composition's short- and long-term impacts on myocardial slice phenotype and function is crucial for establishing standardized, reproducible protocols and reaching a consensus on optimal culturing conditions.

## 4. Current knowledge gaps in LMS culture

Although LMS have been used since 1946,^7^ technical advancements for biomimetic culturing have enabled a novel avenue, the use of chronic, long-term cultured LMS studies. This synergy makes LMS a promising model for bridging the gap between preclinical experiments and clinical trials. However, to fully reach and understand the potential of LMS, there are still several knowledge gaps that need to be addressed.

Notably, it is unclear how well LMS reflect the individual patient characteristics, such as underlying disease etiology, genetic background, co-morbidities, and medication history. This inherent biological variability, poorly explored till now, can introduce potential confounding factors, underscoring the need for stringent protocols to thoroughly document patients’ medical history. A good mitigating practice is ensuring a uniform distribution of patients across experimental groups, for example, an equal representation of etiologies, medication history, etc. across groups. Additionally, we strongly advocate the existence of internal controls, that is, that some LMS from a patient are used for testing the experimental condition while others are allocated to the respective control. This practice prevents bias but also allows for the discovery of patient-specific responses. Importantly, there might be particular research questions that require a specific patient subgroup, further highlighting the need for reliable medical recording and patient recruitment.

Of note, LMS can also be used to study experimental conditions in a heterogeneous population, that more closely mimics the clinical setting, contrary to current preclinical models. However, achieving meaningful results will depend on the production of LMS from a large number of patients and healthy subjects, only possible via a long-term collaboration of multiple academic hospitals and medical centers with cardiothoracic surgery units.

Additionally, while changes between culture systems have been reported,^16,20,25^ the translational potential of LMS has been only hinted at by comparing basic transcriptional changes to freshly produced slices.^18,20,61^ To fully evaluate the translational potential, an in-depth characterization of functional, metabolic, and epigenetic changes is necessary. Studies involving LMS typically follow one of two lines of thought: (i) those conducted acutely after processing or after a short adaptation period^16,61^ or (ii) those executed after a 2–3 weeks of functional stabilization.^20,25^ While these approaches have not been comprehensively compared, the choice between these two approaches may depend on the specific research question. In some cases, it may be desirable to challenge LMS as early as possible, in a period when the tissue still has not yet adapted to culture conditions and preserves the *in vivo* properties to the fullest extent. On another note, chronic studies, or those involving response to pharmacological compounds, may require stable baseline conditions, and can only be achieved after several weeks in culture. Nonetheless, for drawing conclusions with translational validity, it is important to investigate in more depth what changes occur in culture to accurately interpret the relevance and applicability of the findings to the *in vivo* situation. *Table 2* summarizes and provides practical suggestions linked to the use of LMS.

**Table 2:** Practical suggestions for the use of LMS

Aspect	Guidelines
Ethical considerations	Obtain necessary ethical approvals Comply with institutional and national guidelines
Patient data reporting	Report patient age, sex, and specify LVAD status; if present, report its duration Specify the tissue's originating procedure and sample location within the heart Document the etiology of heart disease, medications, and relevant co-morbidities
Tissue procurement	Minimize warm ischemia time during sample procurement (ideally < 5 min) Preferably flush the heart with cold cardioplegia before removal to preserve tissue viability optimally
Timing of LMS use	Conduct studies acutely for better clinical representation Conduct studies after stabilization if a stable baseline is needed (particularly relevant for chronic studies) Consider the duration of culture stability and LMS rundown, which varies according to the species, in experimental design human (stable: at least up to 4 months, no significant rundown)^20^; non-human primates (stable: day 3 to day 10, max. longevity 21 days)^57^; pig (stable: day 3 to day 6, max. longevity 2–3 weeks)^80^; mouse (stable: up to 7 days, max. longevity 7 days)^25^
LMS preparation	Generating LMS has a steep learning curve, and expertise in the processing highly impacts the success rate Keep the cardiac tissue submerged at approximately 4°C and process it as quickly as possible Mount tissue with the epicardium facing down for minimal myofiber cross-section Maintain slice thickness at 300 μm; thinner slices can be generated when the visualization of sarcomeres is needed The working conditions should be sterile Indicate vibratome model and blade reference. Preferably use a vibratome that allows for z-axis alignment and calibrate it <1.0 μm. Vibratome settings: amplitude of 1–2 mm, vibrating frequency of 70–80 Hz, and an advance rate of 0.03–0.07 mm/s
Culture conditions	Be aware that removal of pathological stimuli may induce remodeling and loss of pathologic traits Avoid overloading slices unless required for the experimental setup (*e.g.* inducing hypertrophic response) Avoid loading slices below physiologic levels as this will lead to atrophy and dedifferentiation. Limit the use of serum, except when strictly necessary for the experimental design, due to reproducibility challenges and ethical concerns, prefer chemically defined, serum-free media
Readouts and measurements	Select readouts based on study objectives but always test experimental and control conditions on LMS derived from the same patient When subjecting LMS to multiple consecutive treatments, first test the chronic effect of a single treatment Always measure viability or a derivative (*e.g.* contractile force)
Data reporting	Provide detailed methodologies and protocols Distinguish between biological replicates (*i.e.* number of patients) from technical replicates (*i.e.* number of slices from the same heart subjected to the same condition) Use a minimum of 3 biological replicates and 3 technical replicates to ensure meaningful data interpretation (*N.B.* the research question influences the statistical approach (*e.g.* randomization model vs. population model^97^) and thereby the number of required replicates. For treatments with small effect sizes or heterogeneous patient subsets, technical and biological replicates might need to be increased) Use normalization strategies to reduce biological and slicing-induced variability between LMS Report all experimental conditions transparently

A major challenge in LMS research is the limited access to fresh human tissue. This can be addressed through two approaches: preservation pre- or post-slicing and the usage of small cardiac biopsies. Hypothermic preservation of cardiac biopsies pre-slicing for up to 36 to 55 h is compatible with the generation and maintenance of LMS^20,89^ but the limited sample size and scope of existing studies prevent a comprehensive understanding of the effects of hypothermic preservation. Moreover, hypothermic preservation of LMS for up to 6 days post-slicing has been reported, with overall conservation of myocardial structure but with an increase in field potential duration.^79^ Optimization of hypothermic and even cryogenic preservation could expand access to fresh tissue for LMS research.^90,91^ Recently, a study reported the successful preparation of LMS from previously cryopreserved myocardial biopsies, thereby extending the interval between tissue collection and LMS preparation.^40^ However, it is worth noting that even though cytomorphology and cell-cell connections seemed undisturbed in LMS prepared from cryopreserved biopsies, no functional assessments were provided.^40^ To the best of our knowledge, no studies have yet explored the direct cryopreservation of LMS. Alternatively, utilizing small cardiac biopsies^35,36^ or mini-LMS^92^ is an exciting avenue, as it increases the availability of human cardiac tissue, enables higher throughput of LMS, allows for models of personalized medicine, and allows for providing insights from LMS at different time points (*e.g.* throughout different stages of disease progression). However, the current use of LMS derived from biopsies is limited to short-term measurements. Adapting biomimetic culture systems to fit much smaller LMS could overcome this limitation and simultaneously increase throughput, unlocking the full potential of biopsy-derived LMS. While LMS share limitations with papillary muscle and trabeculae preparations, such as variability, limited tissue accessibility, and a lack of healthy control samples, their higher throughput and extended culture longevity with continuous functional characterization provides a unique advantage in bridging gaps within the current drug development pipeline.

## 5. Reporting and analysis

An important consideration when presenting data obtained from LMS is linked to data reporting and the choice of disclosing normalized or absolute values. A detailed description of the variety of LMS measurements and associated experimental considerations can be found in Watson *et al.*^93^ As discussed above, the properties of myocardial slices vary according to the species, intra-cardiac origin, and transmural localization.^94–96^ In addition, and despite one's best efforts to keep methodological variance to a minimum, minor differences between successive slices can still occur (*e.g.* presence of fibrotic tissue or large intramyocardial vasculature, variations in slice thickness and the cross-sectional area), intrinsically linked to the manual trimming of the myocardial transections obtained with a high-precision vibratome. As a result, it is reasonable to expect that parameters like the LMS’ functional properties or metabolic status may vary slightly from slice to slice and that culture-induced remodeling, occurring during the conduction of chronic experiments, might amplify these differences. Hence, reporting and comparing absolute values obtained from different slices can be misleading and create a dependence on larger sample sizes. Yet, the additional reporting of absolute values is desirable as a measure of quality and standardization.

To circumvent these limitations, a common practice across studies is the allocation of slices to a given treatment by taking into consideration its localization in the transmural axis. This process of randomization ensures that the same experimental condition is tested in slices originating from different regions within the cardiac wall, minimizing variability between groups and increasing the robustness of the findings. If real-time data is generated, randomization by allocating different treatments to slices that show similar performance levels (*e.g.* contractile force, diastolic tension) can be an alternative strategy.

Because of the intrinsic variability between slices and to reduce bias, results obtained from slices are typically subjected to normalization, with different strategies having been proposed so far. In several reports, the maximum contractility (*i.e.* maximum contraction force) produced by slices was normalized to their respective cross-sectional area.^16,18^ The rationale for such normalization is that a bigger slice will possess more force-producing cardiomyocytes and, therefore, contract more strongly than a smaller counterpart. However, the force produced by a slice is not a function of the surface area or absolute number of cardiomyocytes, but instead by the aggregated short-axis cardiomyocyte cross-section, composed of sarcomeres organized in parallel to the longitudinal plane of the LMS. Hence, the width and thickness, rather than the length of the slice, influence contractility the most, and, as such, maximum force production should be normalized to the product between thickness (normally 300 μm) and the width of the slice. The viability of myocardial slices, assessed by the conversion of MTS tetrazolium into a colored formazan product by viable cells, was normalized to the slice mass.^16^ Whenever the same parameter is evaluated repeatedly over time (*e.g.* longitudinal studies), it is common to observe a normalization to baseline levels and the display of results as percent change. This has been the case for the determination of viability,^18^ contractility and relaxation,^25,55,80^ and metabolic activity.^18^ Of note, one must ensure that a sufficiently large number of controls is included in the study design to account for rundown as a confounding factor.

All of the above strategies deal effectively with the high variation between individual slices, and it is our opinion that they are essential for a more comprehensive understanding of a given experimental condition. Despite little progress has been made, most likely due to technical limitations (*e.g.* penetrance of viability dyes across the slice), it would be valuable to integrate the number of viable cells or cellular ratios within a slice for more accurate normalization of data.

## 6. Conclusion

The synergy of LMS and biomimetic culture systems allows for bridging the gap between *in vitro* and *in vivo* experiments. However, employing this sophisticated model requires careful consideration of potential needs and limitations. An overview of the advantages, disadvantages and limitations of the LMS model is provided in *Table 3*. Understanding the alterations arising during specific culture conditions and their implications for research is paramount, yet currently underexplored. Additionally, in LMS studies using human tissue, potential confounding factors such as co-morbidities, medication, and etiology warrant attention. This can be partly mitigated by randomizing slices from the same patient between treatment groups and ensuring robust effects across slices. Normalization strategies are essential for accurate data reporting and to minimize the effects of inter-slice variability.

**Table 3:** Compilation of the advantages, disadvantages, and limitations associated with the use of LMS

Category	Advantages	Disadvantages and limitations
Tissue collection	Enables patient-specific studies Reflects pathological remodeling	Relies on fresh myocardial tissue availability Dependent on patient consent and ethical approval
Slice preparation	Maintains tissue structure and cellular composition Preserves myocardial function and electrophysiology	Requires acquisition of expensive equipment (*e.g.* vibratome, biomimetic culture systems) Acute processing imposes time pressure on personnel
Culturing and maintenance	Biomimetic cultures enable chronic studies (> 4 months) Compatible with testing of electrical and mechanical stimuli	Lacks coronary perfusion, likely inducing vascular remodeling Oversimplified culture conditions lead to tissue adaptation
Experimental applications	High translatability to human physiology Allows real-time data collection Enables comparative and cross-species studies Mid-to-high throughput potential	Genetic manipulation is challenging and heterogeneous Field is young and abundant with inconsistent methodologies
Data analysis and interpretation	Provides insights into patient-specific and disease-specific mechanisms Facilitates studies in heterogeneous populations	Limited complexity compared to whole-heart studies and lacks systemic effect High variability between patients and slices

Ultimately, LMS generated from different species may require distinct optimization to ensure appropriate culture conditions. Collaborative efforts to standardize methodologies and reporting practices will be crucial in maximizing the translational potential of LMS. Maintaining and studying human cardiac tissue *ex vivo* represents a significant advancement in cardiovascular research, bridging the gap between traditional *in vitro* and *in vivo* studies. Although LMS are still in their early stages, this *ex vivo* platform holds great promise for advancing preclinical research, with the potential to refine drug development and enabling more physiologically relevant disease modeling. With continued refinement and standardization, LMS have the potential to significantly impact the future of cardiovascular therapeutics.

## Data Availability

No new data are associated with this review article.
